# P-325. Lingering Effect of the COVID-19 Pandemic: Unnecessary Glove Use by Medical Trainees at a Large Academic Medical Center

**DOI:** 10.1093/ofid/ofae631.528

**Published:** 2025-01-29

**Authors:** Breanna Brown, Dawn Nolt, Judith A Guzman-Cottrill

**Affiliations:** Oregon Health And Science University, Portland, Oregon; Oregon Health and Science University/Doernbecher Children’s Hospital, Portland, OR; Oregon Health & Science University, Portland, Oregon

## Abstract

**Background:**

In 2023, medical students and house staff were observed wearing disposable gloves during pediatric patient encounters, including those in standard precautions. When asked about their rationale for glove use, students and house staff assumed that universal gloving was a component of standard precautions. We sought reasons for universal glove use, and if universal gloving for all patient care was followed beyond the Children’s Hospital.

**Figure:**
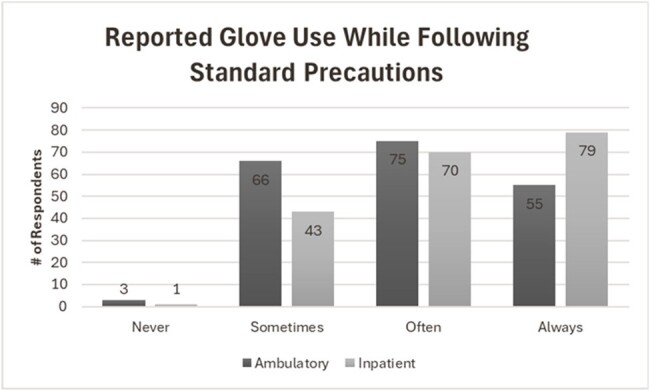

**Methods:**

Residents and fellows in all training programs at Oregon Health & Science University (OHSU) were surveyed. We created an electronic survey including questions about demographics, training level during the pandemic, hand hygiene habits, and facilities’ gloving policies where they worked during the pandemic. A free-text response was available to detail routine glove use. The survey was e-mailed by the Graduate Medical Education office in February 2024 and was open for one month. A reminder e-mail was sent 2 days before closure. The OHSU IRB reviewed the survey and determined it not to be human research.

**Results:**

The survey was sent to 972 trainees, representing 95 programs. The response rate was 23% (n=226); not all questions required responses. Only 30 (25%) were pediatric trainees. In ambulatory settings, 3 (1%) respondents report never gloving, 66 (33%) sometimes, 75 (38%) often, and 55 (28%) always wear gloves as part of standard precautions. In inpatient settings, 1 respondent never wears gloves, 43 (22%) sometimes, 70 (36%) often, and 79 (41%) always wear gloves as part of standard precautions. Of the respondents in medical school during the pandemic: 64 (46%) were taught to wear gloves during all patient care, and 28 (20%) reported hospital policy requiring universal gloving. For those in residency and/or fellowship during the pandemic: 74 (55%) were taught to wear gloves during all care, and 17 (13%) reported policies that required gloving.

**Conclusion:**

Many trainees were taught universal gloving during the pandemic. These habits have continued. The concepts behind standard and transmission-based precautions must be highlighted during medical training. It is imperative that all GME programs educate their trainees about the concepts of standard precautions and appropriate glove use.

**Disclosures:**

**All Authors**: No reported disclosures

